# Proteome profile changes during poly-hydroxybutyrate intracellular mobilization in gram positive *Bacillus cereus* tsu1

**DOI:** 10.1186/s12866-020-01815-6

**Published:** 2020-05-19

**Authors:** Hui Li, Joshua O’Hair, Santosh Thapa, Sarabjit Bhatti, Suping Zhou, Yong Yang, Tara Fish, Theodore W. Thannhauser

**Affiliations:** 1 Department of Agricultural and Environmental Sciences, College of Agriculture, Tennessee State University, 3500 John A. Merritt Blvd, Nashville, TN 37209 USA; 2 R.W. Holley Center for Agriculture and Health, USDA-ARS, Cornell University, Ithaca, NY 14853 USA

**Keywords:** *Bacillus cereus*, Quantitative proteomics, Poly-hydroxybutyrate, Sporulation, Fermentative growth

## Abstract

**Background:**

*Bacillus cereus* is a bacterial species which grows efficiently on a wide range of carbon sources and accumulates biopolymer poly-hydroxybutyrate (PHB) up to 80% cell dry weight. PHB is an aliphatic polymer produced and stored intracellularly as a reservoir of carbon and energy, its mobilization is a key biological process for sporulation in *Bacillus* spp. Previously, *B. cereus* tsu1 was isolated and cultured on rapeseed cake substrate (RCS), with maximum of PHB accumulation reached within 12 h, and depleted after 48 h. Fore-spore and spore structure were observed after 24 h culture.

**Results:**

Quantitative proteomic analysis of *B. cereus* tsu1 identified 2952 quantifiable proteins, and 244 significantly changed proteins (SCPs) in the 24 h:12 h pair of samples, and 325 SCPs in the 48 h:12 h pair of samples. Based on gene ontology classification analysis, biological processes enriched only in the 24 h:12 h SCPs include purine nucleotide metabolism, protein folding, metal ion homeostasis, response to stress, carboxylic acid catabolism, and cellular amino acid catabolism. The 48 h:12 h SCPs were enriched into processes including carbohydrate metabolism, protein metabolism, oxidative phosphorylation, and formation of translation ternary structure. A key enzyme for PHB metabolism, poly(R)-hydroxyalkanoic acid synthase (PhaC, KGT44865) accumulated significantly higher in 12 h-culture. Sporulation related proteins SigF and SpoEII were significantly higher in 24 h-samples. Enzymes for nitrate respiration and fermentation accumulated to the highest abundance level in 48 h-culture.

**Conclusions:**

Changes in proteome of *B. cereus* tsu1 during PHB intracellular mobilization were characterized in this study. The key enzyme PhaC for PHB synthesis increased significantly after 12 h-culture which supports the highest PHB accumulation at this time point. The protein abundance level of SpoIIE and SigF also increased, correlating with sporulation in 24 h-culture. Enzymes for nitrate respiration and fermentation were significantly induced in 48 h-culture which indicates the depletion of oxygen at this stage and carbon flow towards fermentative growth. Results from this study provide insights into proteome profile changes during PHB accumulation and reuse, which can be applied to achieve a higher PHB yield and to improve bacterial growth performance and stress resistance.

## Background

*Bacillus cereus* is a gram-positive, facultative anaerobic bacterium that is widely found in soil and other environments. This species of bacteria can grow efficiently by assimilating a wide range of carbon sources including glucose, sucrose, glycerol, oil fat among others [1]. The bacterial strain was reported to produce poly-hydroxyalkanoates (PHAs) as high as 80% of cell dry weight [2]. PHAs are a class of aliphatic polyesters produced by a large number of bacteria and they are used as a reservoir of carbon and energy [3]. Poly-3-hydroxybutyrate (PHB) is the first and most well characterized member in the PHA family. Since its first discovery in 1925, more than 100 polymer structures with different physical properties have been identified in the PHA family [4]. The PHA-derived plastics is characterized as biodegradable, biocompatible, water insoluble, oxygen permeable and high-temperature resistant, and thus it has become a promising substitute for petroleum-based plastics [5]. However, the high production cost of PHAs bioplastics remains a major limitation for its commercialization and industrialization [6].

PHA polymers are accumulated and stored as intracellular granules when bacteria are growing under excess carbon supply condition. Bacteria can utilize products from metabolic pathways such as acetyl-CoA (from glycolysis), enoyl-CoA (from fatty acid β-oxidation) and (R)-3-hydroxy-acyl-ACP (from fatty acid de novo synthesis) to synthesize PHAs [7, 8]. The most prevalent PHB biosynthesis process starts with acetyl-CoA and goes through condensation, reduction and polymerization catalyzed by acetyl-CoA acetyltransferase (PhaA), acetoacetyl-CoA reductase (PhaB), PHA synthase (PhaC) respectively for the final PHB production. In another common pathway, enoyl-CoA from fatty acid β-oxidation is first oxidized to (R)-3-hydroxyacyl-CoA by (R)-specific enoyl-CoA hydratase (PhaJ) before polymerized into PHB polymer by PhaC [9]. Genes involved in the PHB biosynthesis pathway are co-localized in the chromosome region and organized in an operon.

Bacteria can mobilize PHB polymer when faced with carbon-limited conditions. During this process, the PHB polymer is first depolymerized into its monomer by PHB depolymerase (PhaZ). Previous research was able to detect (S)-3-hydroxybutyryl CoA, crotonyl-CoA and acetyl-CoA as PHB degradation intermediates, which links PHB mobilization with other primary and secondary metabolic pathways like glycolysis, β-oxidation, and TCA cycle [10]. However, mechanisms for reutilization of PHB is not yet fully understood.

In *Bacillus* spp., another key feature is the sporulation process which produces dormant endospores under nutritional stresses [11]. Even though, a variety of self-rescue mechanisms are triggered by starvation, such as the activation of chemotaxis proteins, secretion of hydrolytic enzymes to recycle extracellular energy, sporulation is considered to be the ultimate response [12]. In response to the nutrient depletion as a signal for endospore formation, PHB degradation occurs. In culture, PHB reaches the highest level of accumulation before the formation of fore-spore structure and starts degrading during endospore maturation [13]. More evidences of PHB being mobilized for sporulation came from a study showing that the deficiency in PHB production was associated with reduced spore formation, and the supplementation of exogenous fatty acids was able to recover this sporulation process [14].

When encountering anoxic conditions, many *Bacillus* spp. are capable of using nitrate as electron acceptor, where the various substrate dehydrogenases transfer electrons to the acceptor reductases (nitrate and nitrite reductases). In these *Bacillus* spp., nitrate reduction is always coupled with the fermentative growth, during which ATP is produced by the conversion of pyruvate and acetyl-CoA to a range of fermentative end-products (lactate, acetate, ethanol) [15–17]. Dissolved oxygen (DO) level is an important factor in PHB production using *Bacillus* spp. A higher PHB yield can be achieved by reducing DO from 40 to 20%, whereas severe PHB degradation was observed when DO further dropped down to 5–10% [18]. Consequently, DO level in culture can determine either the shift of acetyl-CoA towards PHB synthesis or the degradation of stored PHB to feed into other metabolic pathways.

Previously, our lab reported isolation of *B. cereus* tsu1, the genome was predicted to have 5763 proteins (NCBI accession no. JPYN01; https://www.ncbi.nlm.nih.gov/Traces/wgs/JPYN01?display=proteins&page=1) [19]. In this study, *B. cereus* tsu1 was cultured on rapeseed cake substrate (RCS) without additional supplements as described before [20]. When examined under microscope, the maximum of PHB accumulation was reached within 12 h (before stationary phase) and it was nearly depleted in 48 h (when mature endospores were released). A quantitative proteomic analysis during these processes was performed to identify the proteomic profile changes of *B. cereus* tsu1 for a better understanding of PHB mobilization mechanisms and to discover strategies in improving bacterial growth performance to achieve a higher PHB yield.

## Results

### Growth phases and PHB intracellular mobilization of *B. cereus* tsu1

**Fig. 1:**
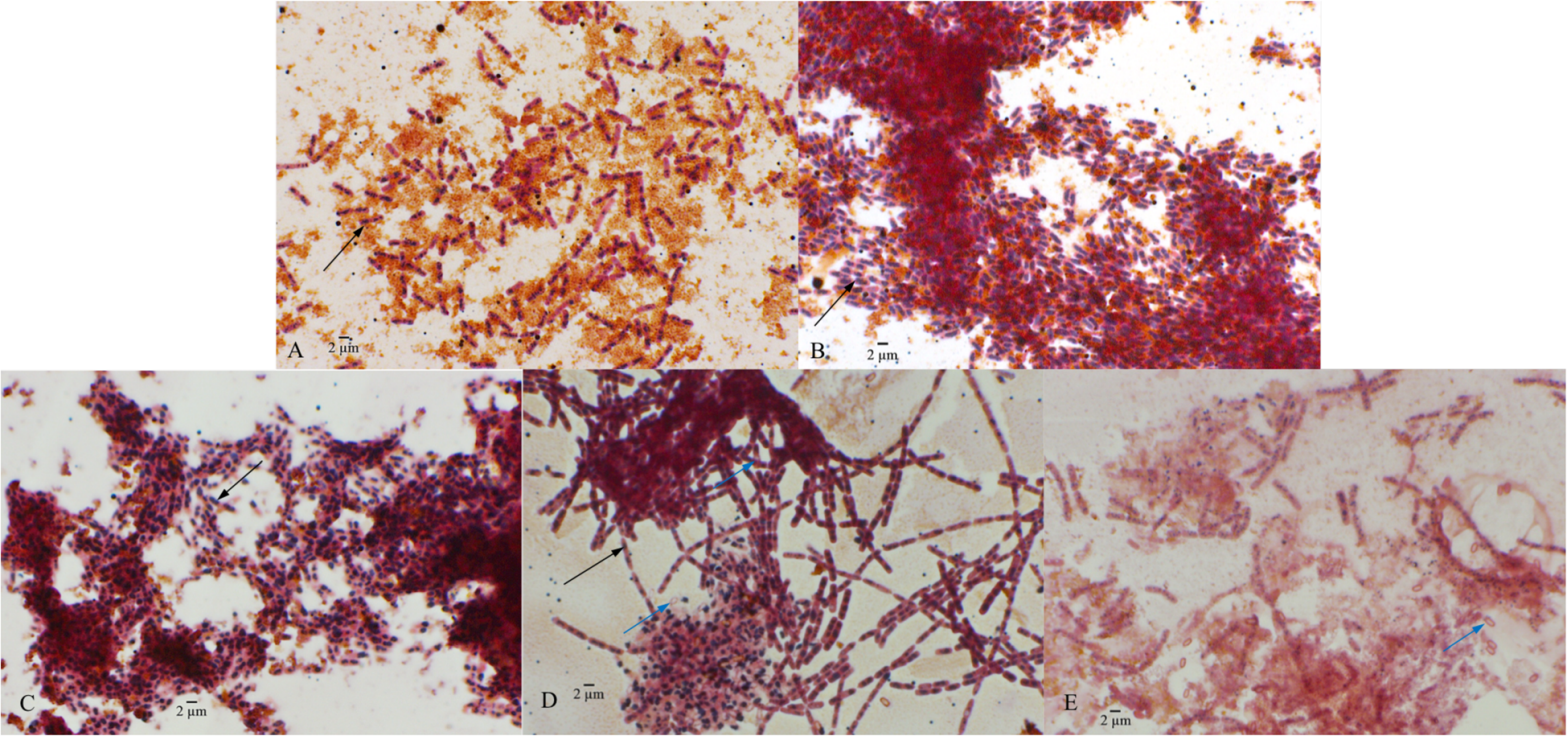
PHB accumulation status in *B. cereus* tsu1. *B. cereus* tsu1 was cultured in RCS medium for 6 h (**a**), 9 h (**b**) and 12 h (**c**), 24 h (**d**), 48 h (**e**). In RCS medium, PHB accumulation was observed at an early stage (6 h) and reached highest accumulation before 12 h. Fore-spore and spore structure were observed at 24 h, significant PHB degradation was observed at 48 h. (Black arrow indicates PHB; blue arrow indicates fore-spore and spore)

*Bacillus cereus* tsu1 was cultured using RCS medium and cells were stained with Sudan black to observe PHB accumulation status (Fig. 1). In 6 h-culture, PHB granules were observable but smaller in size. In 9 h-culture, the granules aggregated and formed clusters, and reached the highest accumulation before 12 h. Bacterial cells were collected at 12 h, 24 h, 48 h and stored at − 20 °C for protein extraction. The reasons for selecting these three time points were 1) bacterial cells in 12 h-culture were loaded with PHB when examined using the Sudan black staining method; 2) in 24 h-culture, most cells were still filled with PHB, but some cells were sporulating with fore-spore, and spore structure visible under the microscope; 3) significant degradation of PHB was observed in 48 h-culture, and even though some mature endospores were released, most cells were still in vegetative state.

### Quantitative proteomic profile and identification of significantly changed proteins

**Fig. 2:**
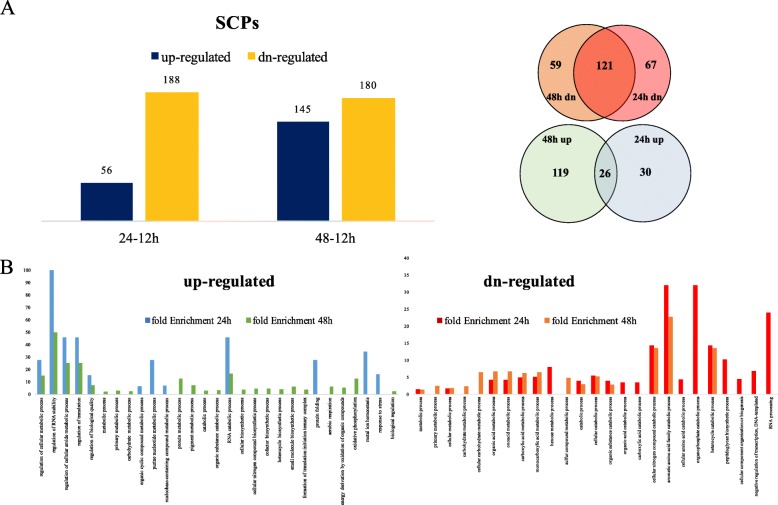
Significantly changed proteins (SCPs), and gene ontology classification using the PANTHER classification system. **a**: SCPs were compared between 24 h- and 12 h-cultures (56 up-regulated proteins, 188 down-regulated protein), between 48 h- and 12 h-cultures (145 up-regulated, 180 down-regulated); Venn diagram shows common and unique SCPs in 24 h–12 h and 48 h–12 h samples. **b**: Gene ontology classification. The enriched biological processes in 24 h:12 h up-regulated SCPs (blue), 48 h:12 h up-regulated SCPs (green), and 24 h:12 h down-regulated SCPs (red), 48 h:12 h down-regulated SCPs (orange) were compared. Proteins assigned to at least one category could be counted more than once

Protein was extracted from 12, 24 and 48 h bacterial cultures in RCS medium. For quantitative proteomics analysis, three biological replicates were included for each time point. The nano-LC-MS/MS identified 3215 proteins and 2952 quantifiable proteins which were reported with at least two unique peptides across all the samples (Additional file 1: Table S1). For these quantifiable proteins, the reporter ion intensity of all constituent peptides was log_2_-transformed. The datasets were subjected to a t-test and the adjusted *P* values were obtained after correction by false discovery rate (FDR). Proteins with log_2_ fold ≥1.5 standard deviations and FDR adjusted *p*-value ≤0.05 were listed as significantly changed proteins (SCPs) for each pair of sampling time-points (24 h:12 h, or 48 h:12 h). Protein fold changes were obtained from anti-log conversion of log_2_ fold. When comparing the 24 h- and 12 h-samples, 244 SCPs were identified [FDR < 0.05, and fold changes (24 h/12 h) < 0.76 or > 1.31], including 56 up-regulated and 188 down-regulated proteins. From 12 h- to 48 h-culture period, 325 SCPs passed the thresholds [FDR < 0.05, and fold changes (48 h/12 h) < 0.67 or > 1.50], with 145 up-regulated and 180 down-regulated proteins (Fig. 2 a, Additional file 2: Table S2–1). Results of t-test and FDR analyses using SAS were listed in the Additional file 3: Table S3–1, Table S3–2.

Functional classification of SCPs was performed using the PANTHER classification system (v.14.1). The biological processes enriched only in the 24 h:12 h up-regulated SCPs include purine nucleotide metabolism, protein folding, metal ion homeostasis, response to stress; the 24 h:12 h down-regulated SCPs were classified into processes of carboxylic acid catabolism, cellular amino acid catabolism, peptidoglycan biosynthetic process, RNA process. The 48 h:12 h SCPs were enriched into biological processes including carbohydrate metabolism, protein metabolism, oxidative phosphorylation, formation of translation ternary structure (Fig. 2 b).

### Enzymes for PHB biosynthesis and intracellular degradation

**Fig. 3:**
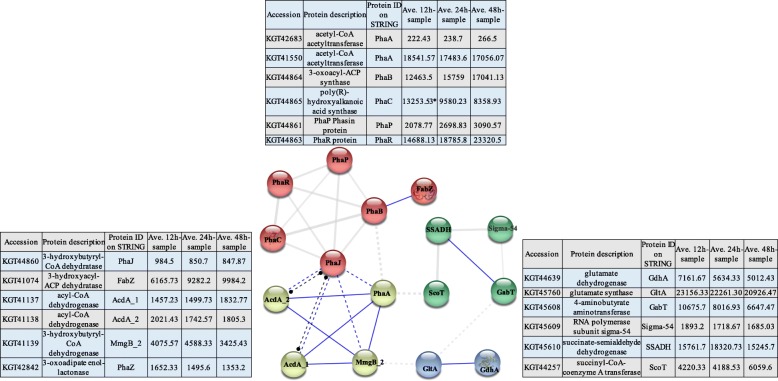
STRING protein interaction network of enzymes for PHB biosynthesis pathways, and protein abundance levels over time. STRING version 10.5 was used to construct protein–protein interaction networks of PHB biosynthesis enzymes in annotated genome of *B. cereus* tsu1. Medium confidence (0.400) was applied and disconnected nodes were hidden. MCL clustering was using inflation parameter 3. Lines between nodes represent their action effects. Enzymes for PHB biosynthesis and their abundance level at three time points are listed in Additional file 2: Table S2–6

The maximum of PHB accumulation in *B. cereus* tsu1 was observed within 12 h of culture on the RSC medium. According to our previous study [20], the genome of *B. cereus* tsu1 was annotated with genes in three different pathways for PHB polymerization. STRING database (version 10.5) of *B. cereus* was used for constructing the protein-protein interaction network of all enzymes in the three PHB synthesis pathways, and protein abundances were compared among the three time points (Fig. 3). The primary pathway starts with acetyl-CoA, using enzymes encoded by a *pha* locus which consists of a *phaR*-*phaB*-*phaC* operon and a *phaP*-*phaQ*-*phaJ* operon in the opposite direction. Poly(R)-hydroxyalkanoic acid synthase (PhaC, KGT44865) had the highest abundance level at early stage of bacterial growth, while the synthase subunit PhaR (KGT44863) displayed an opposite change. PhaR protein was reported as a global regulation factor, with an impact on PHB biosynthesis [21, 22]. Both 3-oxoacyl-ACP synthase (PhaB, KGT44864) and phasin protein (PhaP, KGT44861) accumulated to the highest abundance level at 48 h. PhaQ (KGT44862), a new class of PHB synthesis transcription regulator, was not identified in the proteome analysis. The second pathway uses intermediates of fatty acid β-oxidation as substrates, and reactions are catalyzed by acyl-CoA dehydrogenase (AcdA_1 and AcdA_2) and 3-hydroxybutyryl-CoA dehydratase/enoyl-CoA hydratase (PhaJ). Both AcdA_2 and PhaJ had a higher abundance level at 12 h [23]. The third pathway of PHB synthesis involves utilization of succinyl-CoA from TCA cycle [24], this pathway is catalyzed by SSA dehydrogenase (GabD, KGT45610), 4-hydroxybutyrate dehydrogenase (GabT, KGT45608), and succinyl-CoA-coenzyme A transferase enzyme (ScoT) [7]. Most of these enzymes had a higher abundance level at 12 h. ScoT (KGT44257) is an enzyme associated with both PHB synthesis and consumption; its abundance reached the highest level in 48 h-culture. In the 48 h-sample, PHB was observed to have undergone significant degradation. For PHB degradation, the enzyme 3-oxoadipate enol-lactonase which previously confirmed with PHB intracellular degradation activity in *B. thuringiensis* ATCC35646 [25] was annotated on the *B. cereus* tsu1 genome. Despite of significant PHB degradation observed at 48 h, the abundance of 3-oxoadipate enol-lactonase (KGT42842) for PHB depolymerization was at highest level at 12 h and slightly reduced over time.

### PHB mobilization and related metabolic pathways

**Fig. 4:**
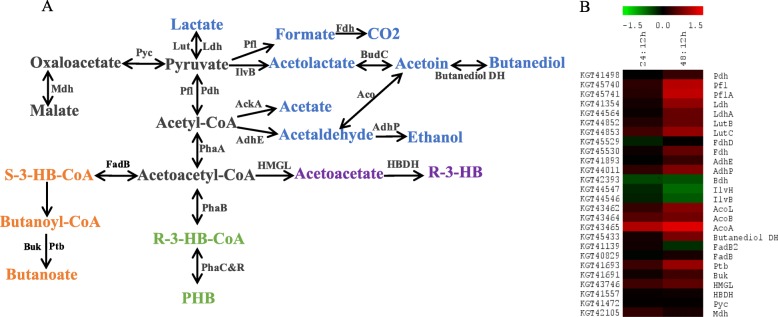
Schematics of pathways related with PHB intracellular mobilization in *B. cereus* tsu1. **a**: Interactions between PHB intracellular mobilization, and other major related pathways. EMP, lipid metabolism, and TCA provide carbon resource for PHB biosynthesis. Pyruvate (anaerobic) fermentation, butanoate metabolism compete carbon resource with PHB synthesis. **b**: Heat map shows log_2_ transformed fold changes of proteins in pathways related with PHB intracellular mobilization

In *Bacillus* spp., PHB formation and mobilization are important metabolic processes interacting with other major pathways. As shown in Fig. 4 a, PHB biosynthesis starts with acetyl-CoA, which participates in several essential biochemical reactions including Embden-Meyerhof-Parnas (EMP) pathway, lipid and protein metabolism, and TCA. PHB mobilization and recycling provide carbon and energy resource for metabolic pathways such as pyruvate fermentation and butanoate metabolism [26].

In this study, most enzymes in EMP, pentose phosphate pathway (PPP), and TCA cycle did not show significant changes among the three time points (Additional file 2: Table S2–2). In EMP, glucose-6-phosphate isomerase (KGT41362) was significantly down-regulated at 0.7 and 0.58 fold in 24 h- and 48 h-cultures. In PPP, 6-phosphogluconate dehydrogenase (KGT42918) was down-regulated by 0.66 fold at 48 h. In glyoxylate shunt bypass of TCA, malate synthase (KGT44986), isocitrate lyase (KGT44987) were down-regulated at 0.61 and 0.66 fold respectively at 48 h.

Butanoyl-CoA converted from acetyl-CoA is another major carbon metabolic product. Using this pathway, bacteria can produce butanoate when grown at neutral pH on glucose [27]. The first step in this pathway is identical with PHB biosynthesis. Afterward, acetoacetyl-CoA is converted to (S)-3-hydroxybutanoyl-CoA by 3-hydroxybutyryl-CoA dehydrogenase (KGT41139). The final two-step conversion of butanoyl-CoA to butanoate provides energy source for cells, as ATP is generated. This two-step conversion process is catalyzed by phosphate butyryltransferase (KGT41693) and butyrate kinase (KGT41691). Phosphate butyryltransferase was up-regulated at 1.86 fold at 48 h, and butyrate kinase had a higher abundance at 48 h compared to the other two time points (Fig. 4 b, Table 1).

**Table 1:** Significantly changed proteins in 24 h:12 h and 48 h:12 h samples related with carbohydrate metabolism, stress, sporulation, and energy metabolism

Function	Accession	Protein description	24 h:12 h FDR adjusted *p*-value	Fold Change (24 h:12 h)	48 h:12 h FDR adjusted *p*-value	Fold Change (48 h:12 h)
Carbohydrate Metabolism	KGT44865	poly(R)-hydroxyalkanoic acid synthase	<.0001	0.75	⎻	⎻
	KGT41362	glucose-6-phosphate isomerase	<.0001	0.70	<.0001	0.58
	KGT42918	6-phosphogluconate dehydrogenase	<.0001	0.70	<.0001	0.58
	KGT44986	malate synthase	⎻	⎻	<.0001	0.61
	KGT44987	isocitrate lyase	⎻	⎻	<.0001	0.66
	KGT41693	phosphate butyryltransferase	⎻	⎻	<.0001	1.86
	KGT45740	formate acetyltransferase	⎻	⎻	<.0001	2.11
	KGT45741	pyruvate formate lyase-activating protein	⎻	⎻	<.0001	2.20
	KGT41354	lactate dehydrogenase	⎻	⎻	<.0001	1.81
	KGT44852	amino acid dehydrogenase	⎻	⎻	<.0001	1.51
	KGT44853	lactate utilization protein C	⎻	⎻	<.0001	1.84
	KGT45530	oxidoreductase	⎻	⎻	<.0001	1.50
	KGT44011	ethanol-active dehydrogenase/acetaldehyde-active reductase	⎻	⎻	<.0001	1.68
	KGT43462	dihydrolipoamide dehydrogenase	⎻	⎻	0.0112	1.72
	KGT43464	pyruvate dehydrogenase	0.0043	1.42	<.0001	1.59
	KGT43465	acetoin:2,6-dichlorophenolindophenol oxidoreductase subunit alpha	<.0001	2.06	<.0001	2.50
	KGT45608	4-aminobutyrate aminotransferase	⎻	⎻	<.0001	0.65
	KGT40985	sigma-54 modulation protein	⎻	⎻	<.0001	1.54
	KGT44547	acetolactate synthase	⎻	⎻	<.0001	0.65
	KGT44564	2-hydroxyacid dehydrogenase	⎻	⎻	<.0001	1.52
	KGT45433	butanediol dehydrogenase	⎻	⎻	<.0001	1.64
	KGT42393	butanol dehydrogenase	<.0001	0.75	⎻	⎻
	KGT42476	dipicolinate synthase subunit A	<.0001	0.68	<.0001	0.57
Stress	KGT43173	glyoxalase	<.0001	0.60	<.0001	0.43
	KGT42737	glyoxalase	<.0001	0.68	<.0001	0.56
	KGT42638	glyoxalase			<.0001	0.64
	KGT44383	glyoxalase	<.0001	0.46	<.0001	0.29
	KGT45443	chemotaxis protein	<.0001	0.76	⎻	⎻
	KGT41216	chemotaxis protein	<.0001	0.70	<.0001	0.56
	KGT43768	activator of Hsp90 ATPase 1 family protein	<.0001	0.75	⎻	⎻
	KGT44005	molecular chaperone Hsp20	<.0001	2.29	<.0001	2.54
	KGT45779	chaperonin	<.0001	1.35	⎻	⎻
	KGT42404	copper resistance protein CopZ	<.0001	1.43	⎻	⎻
	KGT42386	RNA-binding protein Hfq	0.0161	1.46	<.0001	1.89
	KGT44525	flagellar hook protein FlgL	⎻	⎻	<.0001	1.77
	KGT44484	flagellin	⎻	⎻	<.0001	1.61
	KGT45678	molecular chaperone DnaJ	⎻	⎻	0.0003	1.86
	KGT45484	disulfide bond formation protein DsbD	⎻	⎻	<.0001	1.74
	KGT42538	anti-terminator HutP	⎻	⎻	<.0001	1.52
	KGT41365	general stress protein	⎻	⎻	0.0207	1.67
	KGT42051	PhoP family transcriptional regulator	⎻	⎻	0.0025	1.58
	KGT43053	stress protein	⎻	⎻	<.0001	1.59
Sporulation	KGT40972	cell division protein FtsX	<.0001	0.75	⎻	⎻
	KGT41025	peptidase M24	<.0001	0.65	<.0001	0.48
	KGT41076	stage III sporulation protein D	<.0001	0.61	<.0001	0.31
	KGT41078	peptidase M23	<.0001	0.59	<.0001	0.45
	KGT41184	spore gernimation protein GerQ	0.0223	1.42	⎻	⎻
	KGT41268	sporulation protein	0.0017	1.66	⎻	⎻
	KGT41433	cell division protein FtsQ	<.0001	0.61	<.0001	0.54
	KGT41447	DNA-binding protein	⎻	⎻	<.0001	0.48
	KGT41601	sporulation sigma factor SigF	0.0182	1.43	⎻	⎻
	KGT41602	anti-sigma F factor	⎻	⎻	<.0001	0.65
	KGT41714	stage III sporulation protein AH	<.0001	0.72	<.0001	0.64
	KGT41715	stage III sporulation protein AG	<.0001	1.59	<.0001	1.58
	KGT41946	BofC protein	<.0001	0.66	0.0003	0.54
	KGT41948	spore cortex protein	<.0001	0.69	⎻	⎻
	KGT41949	peptigoglycan-binding protein LysM	⎻	⎻	<.0001	0.57
	KGT41988	spore coat protein CotS	0.0103	1.49	⎻	⎻
	KGT42013	LuxR family transcriptional regulator	<.0001	0.66	<.0001	0.48
	KGT42151	spore protein	0.0247	1.60	⎻	⎻
	KGT42445	spore coat protein	<.0001	1.70	⎻	⎻
	KGT42520	cell division protein FtsY	⎻	⎻	<.0001	1.52
	KGT42664	small acid-soluble spore protein Tlp	<.0001	0.72	⎻	⎻
	KGT42674	spore protein P	0.0009	0.62	⎻	⎻
	KGT44175	transition state regulator Abh	0.0352	1.75	<.0001	2.75
	KGT44210	Spore coat protein G	<.0001	1.52	⎻	⎻
	KGT44211	spore coat protein	<.0001	0.75	0.0012	0.61
	KGT44517	flagellar motor switch protein	<.0001	0.75	⎻	⎻
	KGT44707	cell division protein GpsB	⎻	⎻	<.0001	2.01
	KGT44778	spore coat protein	⎻	⎻	<.0001	0.62
	KGT44827	cell division protein FtsN	<.0001	1.49	⎻	⎻
	KGT44878	internalin	<.0001	0.58	<.0001	0.61
	KGT45203	spore protein	0.0067	2.01	0.0189	2.23
	KGT45346	stage V sporulation protein R	⎻	⎻	<.0001	0.66
	KGT45552	acid-soluble spore protein H	0.0041	0.70	0.0193	0.50
	KGT45755	spore protein	⎻	⎻	0.0025	0.45
	KGT45877	sporulation protein	<.0001	0.76	<.0001	0.54
	KGT45955	spore gernimation protein GerD	<.0001	0.64	<.0001	0.47
	KGT45975	protein sspF	<.0001	1.90	0.0014	1.52
	KGT45993	stage II sporulation protein E	0.0233	1.50	⎻	⎻
Energy Metabolism	KGT41105	ATP F0F1 synthase subunit B	<.0001	0.67	<.0001	0.60
	KGT45463	quinol oxidase subunit 2	⎻	⎻	<.0001	1.57
	KGT44670	menaquinol-cytochrome C reductase	0.0042	1.35	<.0001	1.77
	KGT42309	cytochrome D ubiquinol oxidase subunit I	⎻	⎻	<.0001	2.33
	KGT44113	nitrate reductase	⎻	⎻	<.0001	2.47
	KGT44114	nitrate reductase	⎻	⎻	<.0001	1.92
	KGT44115	nitrate reductase	⎻	⎻	<.0001	2.75
	KGT44130	nitrite reductase	0.0004	1.92	<.0001	2.65
	KGT44131	nitrite reductase	⎻	⎻	<.0001	2.48

Accession: Protein accession from NCBI database; Protein description: Protein NCBI description; Fold change (24 h:12 h) and (48 h:12 h) were obtained from anti-log conversion of log_2_ ratios

*Bacillus* spp. can grow by substrate-level phosphorylation/ fermentation under anoxic condition [28]. In *B. cereus* tsu1, formate acetyltransferase (KGT45740) and pyruvate formate lyase-activating protein (KGT45741), which catalyze the reversible conversion of pyruvate into acetyl-CoA using radical non-redox mechanism [29, 30], were up-regulated at 2.11 and 2.2 fold in 48 h-culture (Fig. 4 b, Table 1). Lactate dehydrogenase (KGT41354) catalyzing the interconversion of pyruvate to lactate was up-regulated at 1.81 fold; lactate utilization protein C (KGT44853), L-lactate dehydrogenase complex protein LldF (KGT44852) and formate dehydrogenase (KGT45530) were up-regulated at 1.84, 1.51 and 1.5 fold respectively in the same culture. For alcohol fermentation, acetyl-CoA is first converted to acetaldehyde by acetaldehyde dehydrogenase (KGT41893), and then to alcohol by ethanol-active dehydrogenase (KGT44011), the latter protein was up-regulated at 1.68 fold. The acetyl-CoA hydrolase (KGT44257) catalyzing the reaction producing acetate from acetyl-CoA also had the highest abundance in 48 h-sample.

Acetoin or 3-hydroxybutanoate is another form of carbon and energy storage produced and excreted by bacteria when the pyruvate level is high [31, 32]. It can be used to provide energy to feed into other metabolic pathways at stationary phase [33]. In *B. cereus* tsu1, acetolactate synthase catalytic subunit (KGT44244) and regulatory subunit (KGT44245), acetolactate synthase (KGT44547) and catalytic subunit (KGT44546), acetolactate synthase (KGT45211) were observed with higher abundance level in 12 h-culture (Fig. 4 b). Acetolactate decarboxylase (KGT45212) was not identified in this proteomics analysis. The *acu* operon comprises of acetoin-reuptake enzymes- acetoin dehydrogenase (KGT42181), acetoin utilization protein (KGT42182), histone deacetylase (KGT42183), none of these proteins showed significant changes among the three time points (Additional file 2: Table S2–3). Enzymes in the *aco* operon converting acetoin into acetaldehyde and acetyl-CoA were all up-regulated at 48 h, which include dihydrolipoamide dehydrogenase (KGT43462, 1.72 fold), and acetoin dehydrogenase E1β component (KGT43464, 1.59 fold), acetoin dehydrogenase E1α component (KGT43465, 2.59 fold). R,R-butanediol dehydrogenase (KGT45433) catalyzing the reversible oxidation of 2,3-butanediol to acetoin and the practically irreversible reduction of diacetyl to acetoin was up-regulated at 1.64 fold in 48 h-culture (Table 1) [34].

### Sporulation and stress-induced enzymes

In batch-culture process, bacteria are facing constant stresses such as nutrient depletion and suboptimal pH levels. For gram-positive bacteria like *Bacillus* spp., self-rescue mechanisms under nutrient limitation and environmental stress include induction of chemotaxis protein [35], production of antibiotics [36], secretion of hydrolytic enzymes [37], and finally sporulation. In the 24 h-culture, pre-spore and spore structures were observed; in the 48 h-culture, mature endospores were released, meanwhile significant PHB degradation occurred.

**Fig. 5:**
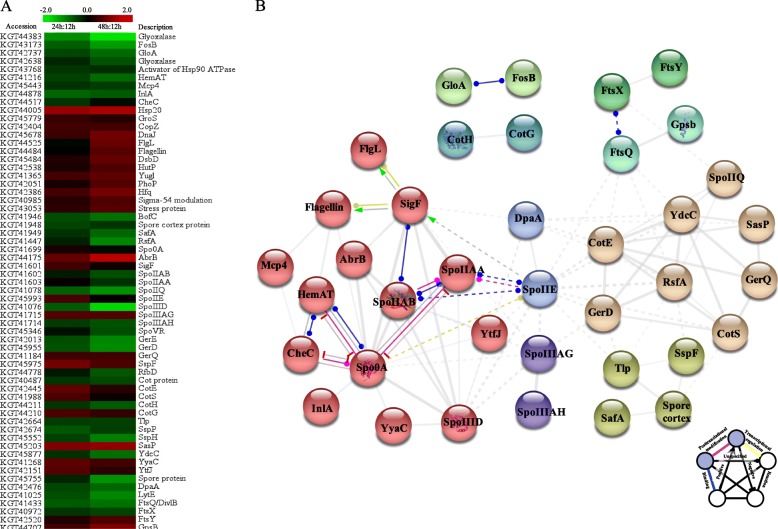
Sporulation-related and stress-induced proteins. **a**: Heat map displays log_2_ fold changes of proteins associated with stress and sporulation annotated on *B. cereus* tsu1. **b**: STRING version 10.5 was used to build protein–protein interaction networks of significantly changed sporulation and stress proteins. The lines in between two nodes indicate the predicted mode of actions. Red nodes represent proteins regulating sporulation process

In the quantitative proteomic analysis of *B. cereus* tsu1, stress related proteins showed with significant changes during culture (Fig. 5 a, Table 1). Glyoxalase/ lactoylglutathione lyase (KGT43173, KGT42737, KGT42638, KGT44383) [38], chemotaxis protein (KGT45443, KGT41216) [39], activator of Hsp90 ATPase (KGT43768) were significantly higher at 12 h (late exponential phase) compared to 24 h (stationary phase). Molecular chaperone Hsp20 (KGT44005), chaperonin (KGT45779) [7], copper resistance protein CopZ (KGT42404), and RNA-binding protein Hfq (KGT42386) [40] were significantly higher at 24 h. Flagellar hook protein FlgL (KGT44525), flagellin (KGT44484), molecular chaperone DnaJ (KGT45678), disulfide bond formation protein DsbD (KGT45484), anti-terminator HutP (KGT42538) [41], general stress protein (KGT41365), PhoP family transcriptional regulator (KGT42051) [42], sigma-54 modulation protein (KGT40985) and stress protein (KGT43053) had the highest abundance level in 48 h-culture.

Thirty-eight proteins related to sporulation were identified with significant changes over time (Fig. 5 a, Additional file 2: Table S2–4). As shown in the proteins interaction network (Fig. 5 b), chemotaxis protein CheY/Spo0A (KGT41699), sporulation sigma factor SigF (KGT41601), anti-sigma F factor (SpoIIAB, KGT41602), anti-sigma F factor antagonist (SpoIIAA, KGT41603), stage II sporulation protein E (SpoII E, KGT45993) are key enzymes involved in sporulation [43, 44]. SigF is the essential enzyme for sporulation induction in *Bacillus* spp*.*. Anti-sigma F factor is the antagonist of SigF, whose activity can be diminished by SpoII E under the regulation of Spo0A [45]. From our results, SigF and SpoII E were up-regulated at 1.43 fold and 1.5 fold, whereas, anti-sigma F factor was down-regulated at 0.65 fold in 24 h-culture. The transition state regulator Abh (KGT44175) acts as a transcriptional regulator during the transition state from vegetative growth to stationary phase and sporulation [46], this protein was up-regulated at 1.75 fold and 2.75 fold in 24 h- and 48 h-cutlures, respectively.

### Aerobic respiration and anaerobic respiration

In aerobic bacteria, oxidative phosphorylation is the major metabolic pathway using carbohydrate oxidation to generate ATP. Most ATP molecules are synthesized by five membrane-bound enzyme complexes (electron transport chain system), which include complex I-NADH: ubiquinone oxidoreductase/ NADH dehydrogenase [47], complex II-succinate-Q oxidoreductase/ succinate dehydrogenase [48], complex III-menaquinol-cytochrome c oxidoreductase, complex IV-quinol/cytochrome c oxidase, and complex V-F0F1-ATPase (Additional file 2: Table S2–5) [43, 49]. Most atp operon proteins had higher abundance at early stage (12 h), and ATP synthase F0 subunits B (KGT41105) was significantly higher in 12 h-culture compared to the other two time points. In 48 h-culture, complex III menaquinol-cytochrome C reductase (KGT44670), and complex IV--quinol oxidase subunit 2 (KGT45463, QoxA), cytochrome D ubiquinol oxidase subunit I (KGT42309, CydA) were significantly up-regulated at 1.77 fold, 1.57 fold, 2.33 fold, respectively.

For *Bacillus* spp., the final electron acceptors can also be nitrate, nitrite, nitrous oxide other than O_2_ when respiration happens under anaerobic condition [50, 51]. In our quantitative proteomics analysis, nitrate reductase NarG, NarH, NarJ, (KGT44113, 44,114, 44,115) were significantly up-regulated at 2.47, 1.92, 2.75 fold; nitrite reductase NirD, NirB (KGT44130, 44,131) were up-regulated at 1.92, 2.48 fold in 48 h-culture (Table 1). These results indicate that, at this time point, the cellular metabolism pathways were changing towards nitrate respiration and fermentation.

## Discussion

In this experiment, growth performance and PHB intracellular mobilization of *B. cereus* tsu1 were examined when rapeseed cake substrate (RCS) was used as the sole nutrients for bacterial culture. In batch culture of *B. cereus* tsu1 in RCS, the maximum PHB was observed within 12 h. Significant PHB degradation occurred as well as release of mature endospores in the 48 h-culture. The bacterial growth performance in RCS was comparable with cultural media such as LB and minimal salt medium M9 supplemented with glucose, while the PHB accumulation occurred the soonest on RCS (data not shown). The quantitative proteomic analysis of *B. cereus* tsu1 used a holistic approach to investigate the entire proteome expressed in bacterial cells at different cell growth and PHB mobilization stages. Totally 3215 proteins were identified, out of which 2952 proteins were quantified in all three time points. Based on PANTHER gene ontology classification, the biological processes enriched with the 24 h:12 h up-regulated SCPs are more related with stress and cell homeostasis, while the 24 h:12 h down-regulated SCPs are associated with cellular catabolic processes. In the 48 h–12 h pair of sample, SCPs were more enriched into carbohydrate/protein metabolism, respiration and energy derivation.

Quantitative proteomics analysis was performed on cultures at distinct stages of PHB accumulation and degradation. Based on differences in protein abundance level between 24 h- and 12 h-samples, enzymes related with PHB biosynthesis, cellular stress and sporulation were identified. Similarly, by comparing between 48 h- and 12 h-samples, enzymes associated with PHB degradation and other metabolic pathways were selected.

Enzymes for PHB biosynthesis and intracellular degradation were quantified in this proteomic analysis, even though, a majority of enzymes were not observed to have significantly abundance changes across the three cultures at 12, 24 and 48 h. PhaC is the key enzyme in PHB polymerization [52] and a significantly higher abundance of this enzyme was observed in 12 h-culture. Accordingly, a shorter interval of sampling strategy should be applied in future studies of these related enzymes. PhaJ is the key enzyme to provide (R)-3HB-CoA monomer for PHB synthesis in the second pathway. Both PhaJ, and enzymes using succinyl-CoA to produce PHB were identified in 12 h-culture, which is an evidence that the bacterium is using all three pathways for PHB accumulation [53, 54]. Additionally, the 3-oxoadipate enol-lactonase catalyzing PHB intracellular degradation showed the highest level in the same 12 h-culture. Taken together, these results indicate that the PHB synthesis and utilization processes occurred simultaneously in these cells. A study on *B. thuringiensis* BMB171 (a mutant used as model organism) showed that PHB degradation remained active even when the gene encoding for 3-oxoadipate enol-lactonase was deleted; these results suggested that additional enzymes are involved in PHB degradation [55]. The 3-oxoadipate enol-lactonase of *B. thuringiensis* contains a lipase box-like sequence (G-W-S102-M-G), and the serine (102) site was proved to be important for the PHB-hydrolyzing activity [25]. The 3-oxoadipate enol-lactonase (KGT42842) was classified as an alpha/beta hydrolase superfamily protein in Protein BLAST on NCBI. To look for other potential proteins contributing to PHB degradation, the alpha/beta hydrolase superfamily proteins on annotated genome of *B. cereus* tsu1 were downloaded, and a multiple sequence alignment was performed to compare sequence homology and to detect putative lipase box-like sequence [56]. As shown in Additional File 4: Fig. S1, alpha/beta hydrolase family proteins KGT43118, KGT41369, KGT41644, KGT42270 were all detected with G-X-S-X-G lipase-box sequence. Bacteria experiencing incomplete PHB mobilization were observed with deficient sporulation and lower stress tolerance. Therefore, enzymes catalyzing PHB degradation may play a crucial role in bacterial survival under unfavorable conditions. The potential PHB degradation enzymes can become targets in future study to increase PHB yield and improve bacterial growth performance.

Spo0A plays a significant role in bacterial sporulation by regulating the activation of SpoII E. The SpoIIE-RodZ complex was reported to have a function in coordinating asymmetric septum formation and SigF activation in *B. subtilis* [57]. Anti-sigma F can bind on SigF and block its ability to form an RNA polymerase holoenzyme (E-sigma F). The function of anti-sigma F can be eliminated by SpoII E [58, 59]. In this study, SpoII E and SigF had a significant up-regulation whereas anti-sigma F with significant down-regulation at 24 h. Nevertheless, the transcription factor Spo0A did not have significant abundance change across the culture period. For many spore forming bacteria, sporulation and PHB degradation seems to occur simultaneously. It was suggested that suppression of sporulation could be used as a strategy to improve PHB yield. However, a mutant *B. thuringiensis* with deletion of *spo0A* was found to be severely impaired in PHB accumulation [45]. Based on these results, it was concluded that the Spo0A transcription factor is required for a global regulation of PHB biosynthesis, sporulation and other cell cycles. As the protein abundance of both SpoII E and SigF was significantly increased during forespore formation, these two proteins can be promising targets in reducing sporulation and improving PHB biosynthesis.

The 48 h-culture was higher enriched with proteins involved in nitrate respiration, which is an indication of oxygen limitation during batch-culture [60]. At this period of time, enzymes for pyruvate fermentation into formate, lactate, ethanol were significantly induced; the abundance of phosphate butyryltransferase (KGT41693) and butyrate kinase (KGT41691) for butanoate biosynthesis reached the highest level [61]; expression of proteins for the acetoin and butanediol metabolism was also highly induced. These pathways utilize the same carbon sources as PHB biosynthesis. The decrease/depletion of the cellular carbohydrate content may induce catabolism of PHB [32]. These results concurred with the significant degradation of PHB in the cells at 48 h.

## Conclusion

Proteome profile changes during PHB intracellular mobilization in *Bacillus cereus* tsu1 was identified in this study. Results revealed: 1) the key enzyme PhaC for PHB synthesis and 3-oxoadipate enol-lactonase for PHB degradation were detected in all samples and both reached a higher abundance in 12 h-culture implying the concurrence of PHB synthesis and utilization at this time point; 2) the protein abundance level of SpoIIE and SigF was significantly increased to induce asymmetric septum formation and sporulation, these two can be promising target genes for delaying sporulation and thus increasing PHB accumulation; 3) when oxygen became limited, enzymes for nitrate respiration and fermentation were induced to compete for the carbon resource with PHB biosynthesis.

PHB production in non-spore forming bacteria can be induced by excess carbon and imbalanced nutrients conditions (depleted nitrogen, phosphorus, and low oxygen); whereas the same condition will lead to sporulation, fermentative growth and PHB consumption in *Bacillus* strains. In this context, results from this study provide insights into the proteome profile changes during PHB accumulation and recycling in *B. cereus* tsu1. The identified proteins (genes) can be targeted for modification to achieve a higher PHB yield and to improve bacterial growth performance and stress resistance in *Bacillus* spp.

## Methods

### Bacterial culture

In our previous research [19, 20], we have reported the isolation and genome analysis of *B. cereus* tsu1. The genome sequence is available in NCBI database under accession No. JPYN01.

*Bacillus cereus* tsu1 was cultured in 50-ml tube containing 20 ml rapeseed cake substrate (RCS, 2.5% aqueous extract). A fresh overnight (16 h) single-colony culture in LB broth was used as inoculum (at 1:100 ratio). Bacterial cultures were agitated at 200 rpm and 30 °C. Cells were taken from the culture at 6, 9, 12, 24, 48 h and stained with Sudan Black to observe PHB accumulation under a microscope equipped with 506 color camera and 63X oil lens (Axioimager M2, Zeiss) [62]. Cell samples were collected at 12, 24, and 48 h by centrifugation at 13,000×g, for 5 min. Triplicate cultures were included for each time point.

### Protein sample preparation and TMT labeling

Proteins were extracted from cell pellets using the SDS phenol based protein extraction method [63]. Briefly, frozen bacterial cell pellets were re-suspended in a buffer containing 2% sodium dodecyl sulfate (SDS), 30% sucrose, 5% β-mercaptoethanol (v/w) prepared in 0.1 M Tris-HCl (pH 8.0) and ground using a Retsch Mixer Mill MM 400 (Retsch GmbH, Germany). Cold phenol was added at 1:1 ratio and the samples were incubated at 4 °C for 2 h. The mixture was centrifuged at 13,000×g 4 °C for 20 min, and protein in the upper phenol phase was precipitated in methanol containing 0.1 M ammonium acetate after overnight incubation at − 20 °C. After a serial of washes in methanol followed by acetone, the air-dried protein pellets were solubilized in 100 mM triethylammonium bicarbonate (TEAB) buffer. Protein concentration was determined using a Qubit Protein Assay Kit (Thermo Fisher Scientific, MA) on a Qubit 3.0 Fluorometer (Invitrogen, CA).

For TMT labeling, protein samples were processed following the instructions in the TMT10plex™ Isobaric Label Reagent Set (Thermo Fisher Scientific). For each sample, 100 μg protein were taken and digested with Sequencing Grade Modified Trypsin (Promega, WI) at 32 °C for 16 h. The three replicates of bacterial samples grown for 12 h were labeled with tags 126, 128C, 129 N; 24 h samples with 127 N, 131 and 129C; and 48 h samples with 127C, 130 N, 128 N [64]. After combining all the labeled samples, SDS and nonionic solvents were removed using Oasis MCX cartridge following the manufacturer’s instructions (Waters; MA). Peptides were eluted in 75% acetonitrile (ACN)/10% NH_4_OH and dried at reduced pressure using a CentiVac Concentrator (labConco, MO). The samples were re-suspended in 100 μl deionized water and re-dried before mass spectrometry analysis.

### hpRP-HPLC fractionation and Nano LC-MS/MS

The high pH reverse phase high performance liquid chromatography (hpRP-HPLC) fractionation and nano liquid chromatography tandem mass spectrometry (nano LC-MS/MS) were carried out following procedures described in the previous research [65]. In hpRP-HPLC fractionation, the TMT 10-plex tagged tryptic peptides were separated into forty-eight fractions using a Dionex UltiMate 3000 HPLC system (Thermo Fisher Scientific) equipped with an XTerra MS C18 column [66]. The forty-eight fractions were further pooled into a total of twelve fractions based on multiple fraction concatenation strategy [67]. All of the twelve fractions were dried and reconstituted in 2% ACN/0.5% FA. For nano LC-MS/MS, an Orbitrap Fusion mass spectrometer (Thermo Fisher Scientific) coupled with an UltiMate3000 RSLCnano system (Dionex, CA) was used to perform the analysis. Briefly, each reconstitute fractions were first injected onto the UltiMate 3000 RSLCnano, which equipped with a PepMap C-18 RP nano trap column (3 μm, 75 μm × 20 mm) for sample on-line desalting, and a PepMap C-18 RP nano column (3 μm, 75 μm × 15 cm) for separation. The Orbitrap was operated in data-dependent acquisition (DDA) mode using fourier-transform mass analyzer with identical settings described in the previous research [65]. MS survey scans were conducted at a resolving power of 120,000 and 50,000 (fwhm) for m/z range of 400–1600 and 105–2000, respectively [68]. All data was acquired under Xcalibur 3.0 operation software and Orbitrap Fusion Tune 2.0 (Thermo Fisher Scientific).

### Protein identification and quantification

All MS/MS raw spectra were processed and database searched using Sequest HT software within the Proteome Discoverer 2.2 (PD 2.2, Thermo Fisher Scientific). *Bacillus cereus* tsu1 protein database (which was constructed using six-frame translation of the assembled genome sequence) was used to search the spectra (database download link: https://www.ncbi.nlm.nih.gov/protein?linkname=bioproject_protein&from_uid=256220). The search parameters, peptide mass tolerance and fragment mass tolerance values were set based on previous research [69]. Identified peptides were filtered for a maximum 1% FDR using the Percolator algorithm in PD 2.2 along with peptide confidence set to high. The TMT10-plex quantification method within PD 2.2 was used to calculate the reporter ratios. Peptide spectra containing all reporter ions were used for downstream quantitation analysis.

### Significantly changed proteins (SCPs) identification, PANTHER and STRING analysis

For protein quantification analysis, it requires that a protein be reported with at least two unique peptides across all biological samples. For these quantifiable proteins, the reporter ion intensity of all constituent peptides was log_2_-transformed. The datasets were subjected to a t-test (General Linear Model) to obtain the log_2_ fold between each pair of time-points, standard deviation and the raw *P* values. The adjusted *P* values were obtained after correction by false discovery rate (FDR). The log_2_ fold of peptides were fitted to a normal distribution, one and half standard deviations (±1.5 SD, i.e., a 90% confidence level) and a FDR adjusted *p*-value ≤0.05 were used as the cut-off threshold values for significantly changed proteins (SCPs) for each pair of sampling time-points (24 h/12 h, or 48 h/12 h). Protein fold changes were obtained from anti-log conversion of log_2_ fold. The statistical analysis was conducted using SAS (version 9.4) [70].

Gene Ontology (GO) functional classification of 24-12 h pair, and 48-12 h pair SCPs was performed using the PANTHER classification system (v.14.1, http://www.pantherdb.org). The gene IDs of SCPs were submitted to the database to carry out PANTHER Overrepresentation test using the PANTHER GO-Slim Biological Process [71]. Proteins assigned to at least one category could be counted more than once. The STRING database (10.5, https://version-10-5.string-db.org) was used to predict protein-protein interaction networks based on certain active interaction sources including textmining, experiments, database, co-expression, neighborhood, gene fusion, co-occurrence [72]. Protein network analysis was performed by submitting SCPs sequences to the STRING database. Medium confidence (0.400) was applied and disconnected nodes were hidden. Protein clusters were created using the Markov Cluster Algorithm (MCL) inflation parameter (MCL = 3). Lines between nodes represent their action effects, while continuous lines representing direct interactions, interrupted lines indicating indirect functional connections. Protein sequences of functionally enriched proteins were subjected to illustrate the possible molecular actions between each other.

## Supplementary information

**Additional file 1 Table S1**. Proteins identified and their normalized abundances in *Bacillus cereus* tsu1 in 12 h-, 24 h-, and 48 h-culture.

**Additional file 2 Table S2–1**. Significantly changed proteins identified in pair of 24 h:12 h and 48 h:12 h. **Table S2–2**. EMP, PP, TCA enzymes and their average abundance in three time-point samples. **Table S2–3**. Enzymes in butanoate and pyruvate anaerobic metabolism and the abundance at three different time points. **Table S2–4**. Significantly changed proteins related with sporulation. **Table S2–5**. Protein abundance of enzymes involved in oxidative phosphorylation and anaerobic/nitrate respiration. **Table S2–6**. PHB biosynthesis and intracellular degradation enzymes and their average abundance in three time-point samples.

**Additional file 3 Table S3–1**. T-test and FDR analysis of 24 h–12 h pair of samples. **Table S3–2**. T-test and FDR analysis of 48 h–12 h pair of samples.

**Additional file 4 Figure S1**. Multiple sequence alignment of A/B hydrolase superfamily proteins on *B. cereus* tsu1 genome.

## Data Availability

The mass spectrometry proteomics data were deposited to the ProteomeXchange database (http://proteomexchange.org/) via the PRIDE partner repository with identifier PXD009960 under project title: TMT-quantitative proteomic study of *Bacillus cereus* tsu1. All other data generated or analyzed during this study are included in this manuscript.

## Abbreviations

PHB: Poly-hydroxybutyrate
RCS: Rapeseed cake substrate
*B. cereus*: *Bacillus cereus*
SigF: Sigma factor F
SpoE II: Stage II sporulation protein E
SCPs: Significantly changed proteins
PHAs: Poly-hydroxyalkanoates
DO: Dissolved oxygen
TMT: Tandem mass tags
FDR: False discovery rate
EMP: Embden–meyerhof–parnas
PP: Pentose phosphate
TCA: Tricarboxylic acid cycle
ATP: Adenosine triphosphate
BLAST: Basic local alignment search tool
*B. subtilis*: *Bacillus subtilis*
*B. thuringiensis*: *Bacillus thuringiensis*
STRING: Search Tool for the retrieval of interacting genes
MCL: Markov cluster algorithm
