# Supplementary material for: Proteome profile changes during poly-hydroxybutyrate intracellular mobilization in gram positive Bacillus cereus tsu1
Source: BMC Microbiol. 2020 May 19;20:122. doi: 10.1186/s12866-020-01815-6 (PMC7236355; doi:10.1186/s12866-020-01815-6)
Supplement: Supplementary file 4 — Additional file 4 Figure S1. Multiple sequence alignment of A/B hydrolase superfamily proteins on B. cereus tsu1 genome. [file 12866_2020_1815_MOESM4_ESM.pdf]

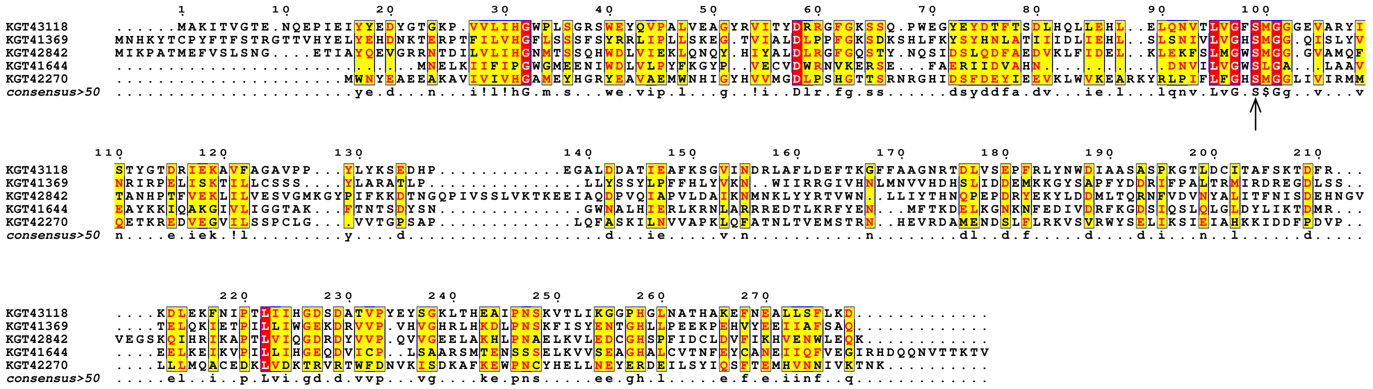

Figure S1. Multiple sequence alignment of A/B hydrolase superfamily proteins on *B. cereus* tsu1 genome.

Multiple sequence alignment of A/B hydrolase proteins were performed using ClustalW followed by online-based ESPript 3.0. The consensus sequences between 3-oxoadipate enol-lactonase (KGT42842) and A/B hydrolase enzymes on *B. cereus* tsu1 genome are highlighted in yellow and pink boxes. The lipase-box like sequence (GXSXG) and the serine site that are important for the PHB-hydrolyzing activity were detected on these A/B hydrolase proteins.
